# Serum osteopontin can improve papillary thyroid cancer risk assessment of Bethesda III thyroid nodules: a preliminary study

**DOI:** 10.1530/EO-21-0005

**Published:** 2021-06-09

**Authors:** T U Kars, M Kulaksızoğlu, İ Kılınç

**Affiliations:** 1 Department of Internal Medicine, Necmettin Erbakan University Meram School of Medicine, Konya, Turkey; 2 Division of Endocrinology and Metabolism Disease, Department of Internal Medicine, Necmettin Erbakan University Meram School of Medicine, Meram, Konya, Turkey; 3 Department of Biochemistry, Necmettin Erbakan University Meram School of Medicine, Meram, Konya, Turkey

**Keywords:** osteopontin, thyroid nodule, thyroid cancer

## Abstract

**Objective:**

Thyroid cancer can be detected in 5–10% of patients with thyroid nodules. Management may be a challenge if fine-needle aspiration biopsy yields Bethesda III findings. Most of these cases undergo surgery and are ultimately found benign. Our aim was to evaluate whether serum osteopontin can accurately estimate thyroid cancer risk in cases with cytologically Bethesda III thyroid nodules and, thereby, decrease the number of unnecessary surgical interventions.

**Design and Methods:**

We obtained blood samples of cases with repeated cytologically Bethesda III thyroid nodules before surgery, and followed up the pathology results after thyroidectomy. We evaluated serum osteopontin from 36 patients with papillary thyroid cancer and compared them with 40 benign cases.

**Results:**

Serum osteopontin levels in patients with papillary thyroid cancer are significantly higher than in benign cases (mean serum osteopontin: 10.48 ± 3.51 ng/mL vs6.14 ± 2.29 ng/mL, *P* < 0.001). The area under the receiver operating characteristics curve was 0.851, suggesting that serum osteopontin could have considerable discriminative performance.

**Conclusions:**

In our preliminary study, high serum osteopontin levels can predict the risk of papillary thyroid cancer in thyroid nodules with Bethesda III cytology. Further studies are necessary to confirm these findings.

## Introduction

Thyroid cancer is the most common cancer of the endocrine system, with a continuously increasing incidence in the last decades (Pellegriti *et al.* 2013). Papillary thyroid carcinoma is the most prevalent histologic type, accounting for approximately 80–90% of all thyroid cancers (Sosa & Udelsman 2006). Thyroid nodules, usually benign, are detected in up to 2–6% of patients on physical exam, 19–68% of patients on ultrasound, and 8–65% on autopsy (Dean & Gharib 2008). Approximately 5–15% of thyroid nodules are malignant (Hegedüs 2004). Thyroid nodules are initially examined by fine-needle aspiration biopsy (FNAB), but the frequent indeterminate or suspicious FNAB results are challenging in terms of defining an appropriate management strategy (Poller & Kandaswamy 2013). When thyroid nodule cytologic results show follicular lesions of undetermined significance or atypia of undetermined significance (FLUS/AUS, Bethesda III), the results are often called indeterminate, and the risk of malignancy reaches 10–30% (Cibas & Ali 2017). Most cases with a cytologic Bethesda III result (confirmed on repeated aspiration) undergo diagnostic thyroid surgery, as molecular tests are still not widely available. However, most patients undergo surgery for an ultimately confirmed benign disease (Yoon *et al.* 2015).

Osteopontin (OPN) is a phosphoglycoprotein rich in sialic acid, expressed in many cells and tissues (Wang & Denhardt 2008, Buback* et al.* 2009). OPN is important for normal biological functioning such as bone remodeling, immunity, and inflammation. It is also involved in the pathophysiology of liver fibrosis, atherosclerosis, and cancer (Coombes *et al.* 2016, Ding *et al.* 2016). Elevated OPN expression was found in many cancers and papillary thyroid cancer (Bramwell 2006, Le 2006, Likui* et al.* 2010, Park *et al.* 2015, Ferreira *et al.* 2016, Wang* et al.* 2018, Zhang* et al.* 2020). In this study, we aimed to investigate the diagnostic and clinical role of serum osteopontin levels in patients who underwent thyroid surgery due to cytologic results repeatedly showing AUS.

## Materials and methods

This study was approved by the Ethics Committee of Necmettin Erbakan University in accordance with the Helsinki Declaration of 1975 (06 May 2016, No. 2016/552), and written informed consent was obtained in all cases. Between May 2016 and April 2017, 120 consecutive cases who underwent diagnostic thyroid surgery after repeated FNAB result showing Bethesda III were included at the university hospital of The Medical School of Necmettin Erbakan University. We did not include cases with other cancers and autoimmune disorder, diabetes mellitus, renal disorder, liver disorder, bone and calcium disorder, thyroiditis findings in ultrasonographic evaluation, or any other inflammatory or medical condition that could influence the parameters under study. Blood samples were obtained before thyroidectomy; 5 mL of venous blood were centrifuged at 4000 ***g*** for 5 min, and the serum was stored at −80°C until further analysis. After surgery, we followed up the pathology results of all included cases. We excluded cases with thyroiditis findings as pathology result. After all exclusions, a total of 103 cases were enrolled in our study. Of those, 65 were benign, and 38 had thyroid cancer. We randomly selected 40 individuals from the benign group due to the limited number of test kits. Of the 38 cancer patients, 36 had papillary thyroid cancer (PTC), one medullary thyroid cancer (MTC), and another follicular thyroid cancer (FTC). To homogenize the thyroid cancer group as PTC, we did not include the two patients diagnosed with MTC and FTC. At last, we evaluated serum samples from 36 PTC patients and compared them with 40 benign cases; we also recorded other data retrospectively from the preoperative evaluation.

After all samples were obtained, serum OPN levels were determined using a Human Osteopontin Platinum ELISA kit (BMS2066, e-Bioscience Inc., San Diego, California, USA). OPN levels were measured by the double antibody sandwich ELISA method according to manufacturer’s instructions.

Statistical analysis was performed using version 22.0 SPSS statistical package. Descriptive findings are shown as mean, standard deviation and minimum–maximum in continuous data, and as numbers and percentages in categorical data. Kruskal–Wallis variance analysis was used for multiple-group comparison of continuous data, and independent-sample *t*-test was used for comparison between binary groups. The *X*^2^ test was used to analyze categorical data, and Pearson correlation was used to analyze the relationship between numerical data. *P*-values ≤0.05 were considered statistically significant. The receiver operating characteristic curve (ROC) was used to analyze the cut-off value of serum OPN levels in both groups.

## Results

**Figure 1:**
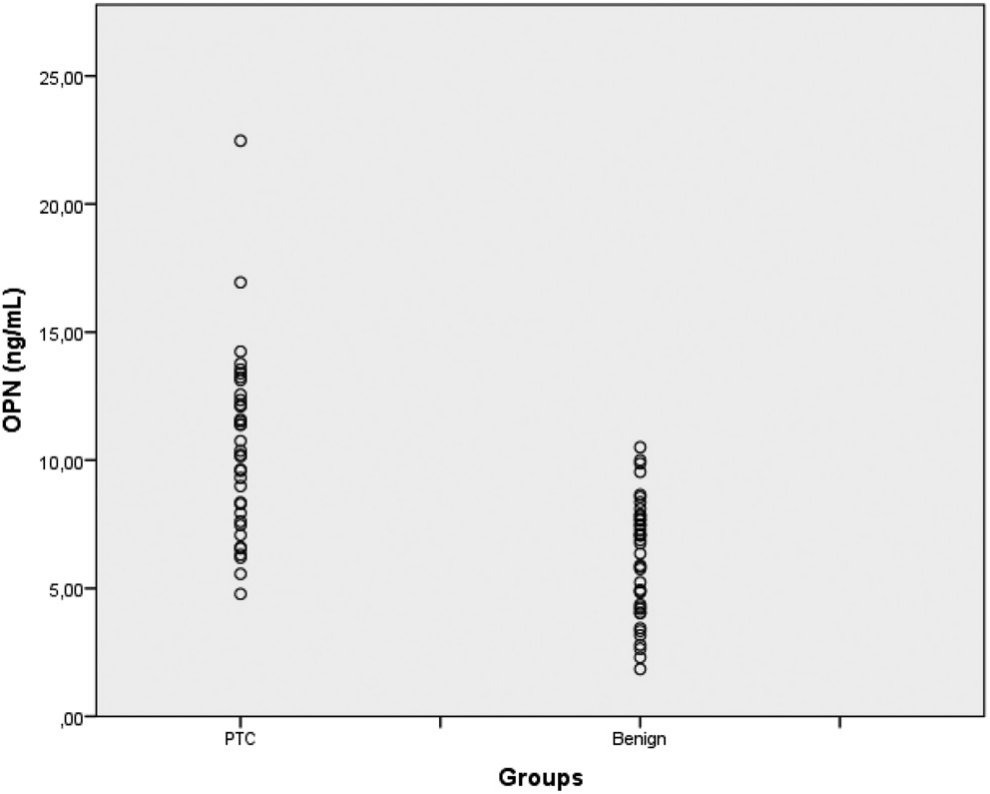
Scattergram presenting the ındividual results of the cases and controls.

Mean age in the PTC group was 44.19 ± 14.25 years. Mean TSH level in the PTC group was 2.16 ± 1.34 µIU/mL, and 1.86 ± 1.06 µIU/mL in the benign group (*P* = 0.292). The analysis of serum OPN levels in PTC patients and benign cases showed significantly higher OPN levels in PTC patients than in the benign group (mean serum OPN level: 10.48 ± 3.51 ng/mL vs6.14 ± 2.29 ng/mL, *P* < 0.001). A scattergram presenting the individual results of patients and controls is shown in Fig. 1.

In the PTC group, we assessed tumor focality; 25 patients (69.4%) were reported as unifocal and 11 patients (30.6%) as multifocal. Twenty-five patients (69.4%) with PTC had no lymphovascular invasion, and 32 (88.9%) had no perineural invasion, while 18 (50%) had capsule invasion. The surgical margin was negative in 31 patients (86.1%). Lymph node metastasis was detected in 9 patients (25%) (Table 1).

**Table 1:** The characteristics of PTC patients and benign cases.

Variables	PTC patients	Benign cases	*P*
Number of subjects (*n*)	36	40	
Male/female (*n*)	7/29	8/32	
Age (year) (mean ± s.d.)	44.19 ± 14.25	46.63 ± 15.9	
TSH (µIU/mL) (mean ± s.d.)	2.16 ± 1.34	1.86 ± 1.06	0.292
OPN (ng/mL) (mean ± s.d.)	10.48 ± 3.51	6.14 ± 2.29	<0.001
Tumor focality (*n*)			
1 Focus	25		
2 Foci	10		
≥3 Foci	1		
Tumor size (*n*)			
Micropapillary (≤10 mm)	11		
Macropapillary (>10 mm)	25		
Lymphovascular invasion (*n*)			
Present	11		
Absent	25		
Surgical margin (*n*)			
Present	5		
Absent	31		
Perineural invasion			
Present	4		
Absent	32		
Lymph node metastasis			
Present	9		
Absent	27		
Capsular invasion			
Present	18		
Absent	18		
TNM stage			
Stages I–II	26		
Stages III–IV	10		
ATA risk classification			
Low risk	12		
Moderate risk	19		
High risk	5		

The PTC group was divided into two groups, micropapillary (≤10 mm) and macropapillary (>10 mm), and the relationship between these groups and serum OPN level examined. Serum OPN levels in the micropapillary group was 10.8 ± 2.38 ng/mL, and in the macropapillary group 10.34 ± 3.94 ng/mL (*P* = 0.668).

To investigate the possible correlation between serum OPN level and tumor aggressiveness in the PTC group, serum OPN level was examined according to tumor focality, TNM staging, ATA risk classification and the presence of lymphovascular invasion, capsular invasion, lymph node metastasis (Table 2). The OPN level in cases of single-focus tumors was 10.28 ± 2.57 ng/mL, and 10.94 ± 5.20 ng/mL in cases of multifocal tumors (*P* = 0.301). In patients with lymphovascular invasion, it was 9.98 ± 3.85 and 10.70 ± 3.41 ng/mL in patients without lymphovascular invasion (*P* = 0.595). In patients with capsule invasion, OPN level was 10.00 ± 3.42 ng/mL, and 10.97 ± 3.64 ng/mL in patients without (*P* = 0.416). OPN level was 9.03 ± 3.71 ng/mL in patients with lymph node metastasis, and OPN level in patients without lymph node metastasis was 10.97 ± 3.37 ng/mL (*P* = 0.340). According to TNM staging, patients of stage 1 (*n* = 25) and 2 (*n* = 1) were considered as thr first group, and patients of stage 3 (*n* = 5) and 4 (*n* = 5) were considered as thr second group, and OPN level was evaluated in these two groups. OPN level was 10.47 ± 3.83 ng/mL in thr first group and 9.90 ± 3.26 ng/mL in the second group (*P* = 0.995). According to the ATA risk classification, 12 (36.3%) of 36 patients with PTC had low-risk, 19 (52.8%) had moderate-risk, and 5 (13.9%) had high-risk. OPN level was 11.5 ± 4.20 ng/mL in low-risk group, 10.09 ± 2.57 ng/mL in medium-risk group and 9.50 ± 4.96 ng/mL in high-risk group. The difference between these groups was not statistically significant (*P* = 0.449).

**Table 2:** Association between serum OPN and clinicopathological characteristics in PTC patients.

Variables	OPN level (ng/mL)	*P*
Tumor focality		0.301
1 focus	10.28 ± 2.57	
≥2 foci	10.28 ± 2.57	
Tumor size		0.668
Micropapillary (≤10 mm)	10.8 ± 2.38	
Macropapillary (>10 mm)	10.34 ± 3.94	
Lymphovascular invasion (*n*)		0.595
Present	9.98 ± 3.85	
Absent	10.34 ± 3.94	
Lymph node metastasis		0.340
Present	9.03 ± 3.71	
Absent	10.97 ± 3.37	
Capsular invasion		0.416
Present	10.00 ± 3.42	
Absent	10.97 ± 3.64	
TNM stage		0.959
Stages I–II	10.47 ± 3.83	
Stages III–IV	9.90 ± 3.26	
ATA risk classification		0.449
Low risk	11.5 ± 4.20	
Moderate risk	10.09 ± 2.57	
High risk	9.50 ± 4.96	

**Figure 2:**
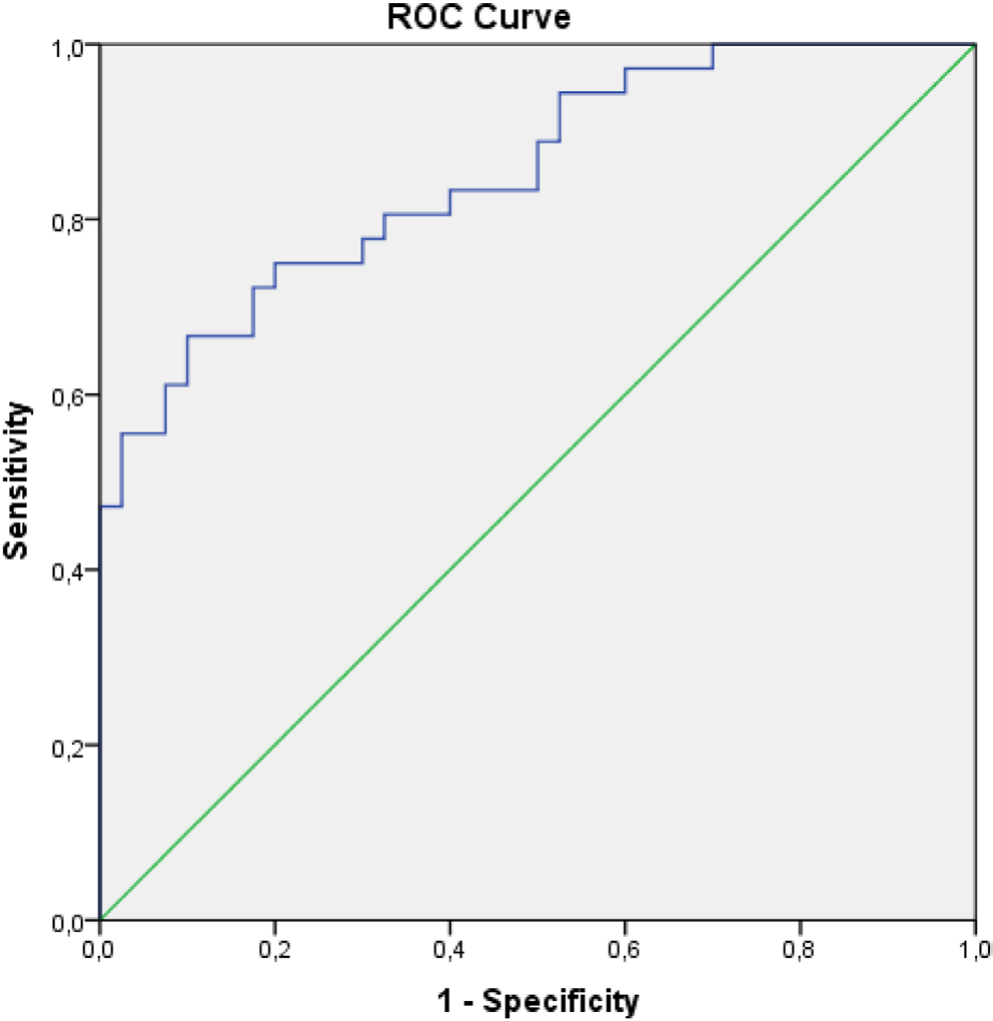
ROC curve analysis for OPN.

In the ROC analysis for the differentiation of patients with PTC from benign cases, the area under the curve for OPN was 0.851 (95% CI; 0.767–0.935). For an OPN cut-off value of 7.90 ng/mL, the sensitivity was 75% and the specificity 80% (Table 3). The positive predictive value was 77.14%, and the negative predictive value 78.04%. ROC curve analysis for OPN is shown in Fig. 2.

**Table 3:** ROC curve parameters of OPN.

	AUC (95%)	Cut-off	*P*	Sensitivity (%)	Specificity (%)
OPN	0.851 (0.767–0.935)	7.90	<0.001	75	80

## Discussion

Thyroid cancer is the most common malignancy of the endocrine system. It accounts for about 90% of endocrine system cancers (Sosa & Udelsman 2006). Since 1990s, the incidence rate of thyroid cancer has been increasing around the world. The increase in thyroid cancer may be primarily due to increased detection of small papillary cancers secondary to more widespread use of neck ultrasonography and FNAB of very small thyroid nodules (Vaccarella *et al.* 2016). FNAB is recommended as a screening tool basically to decide whether a patient requires surgical intervention or can be managed conservatively. There are six major categories of results that are obtained from fine-needle aspiration (FNA), each of which indicates different subsequent management. When thyroid nodule FNA cytologic results show follicular lesion of undetermined significance or atypia of undetermined significance (FLUS/AUS, Bethesda III), the results are often called indeterminate. The percentage of risk of malignancy with Bethesda III is between 10 and 30% (Cibas & Ali 2017). The majority of patients with a cytologic result of FLUS/AUS, confirmed on repeat aspiration, have diagnostic thyroid surgery (usually lobectomy), and almost all of these patients (75–90%) are ultimately confirmed to be benign disease after surgery (Yoon *et al.* 2015). Recently, improvement in the assessment of indeterminate FNAB results with molecular testing reduces the need for diagnostic thyroid surgery. However, molecular tests are not available in most centers. Therefore, it is obvious that there is a need for cost-effective methods to avoid unnecessary surgery.

OPN is a phosphoglycoprotein rich in sialic acid, and is expressed in many cells and tissues, including osteoblasts; osteoclasts; vascular smooth muscle cell; endothelial cells; epithelial cells of the skin, breast, and kidney; and fibroblasts (Wang & Denhardt 2008, Buback *et al.* 2009). OPN is also expressed in immune cells, including lymphocytes; natural killer cells; macrophages; eosinophils; dendritic cells; and microglia (Kawamura *et al.* 2005, Li *et al.* 2017, Morimoto* et al.* 2018). OPN has a physiological functioning such as bone remodeling, immunity, and inflammation (Coombes *et al.* 2016). OPN also has a pathological functioning such as fibrosis; atherosclerosis, and cancer (Ding *et al.* 2016). The role of OPN in tumorigenesis, cancer progression, and survival has been demonstrated in various cancers, including glioblastoma multiforme, hepatocellular carcinoma, colorectal cancer, lung cancer, breast cancer, bladder cancer, melanoma, head and neck squamous cell carcinoma, and acute myeloid leukemia (Bramwell 2006, Le 2006, Conway *et al*.2009, Likui *et al.* 2010, Liersch *et al.* 2012, Güttler* et al.* 2013, Wang *et al.* 2018, Zhang *et al.* 2020). OPN contributes to the malignancy through the promotion of metastasis, maintenance of a stem-like phenotype, epithelial to mesenchymal transformation, activation of cell proliferation pathways, chemotherapeutic and radiation resistance, and interference with immune functioning (Moorman *et al.* 2020).

OPN overexpression in thyroid cancer has also been demonstrated in several studies (Wu *et al.* 2015, Ferreira* et al.* 2016, 2018, Chernaya *et al.* 2018, Wang* et al.* 2020). However, OPN expression was examined only in pathological tissue in most of these studies. In a study, Park* et al.* found that plasma OPN levels were significantly higher in PTC patients than in healthy controls. They also found that plasma OPN, tissue OPN mRNA, and tissue OPN protein levels were significantly lower in patients with PTC and Hashimoto‘s thyroiditis (HT) than in those with PTC alone (Park *et al*.2015). However, the association between thyroiditis and OPN is controversial. Cheng* et al.* found no significant difference in OPN levels between inactive HT and normal controls and between active HT and controls, but they found higher serum OPN levels in active than in inactive Graves‘ disease and controls (Cheng *et al.* 2019). Due to this controversy, we excluded patients with thyroiditis findings in ultrasonographic evaluation from the study. In our preliminary study, we investigated serum OPN level in cases who underwent diagnostic thyroid surgery after repeated FNAB results showing Bethesda III. Our data show that serum OPN level in the PTC group is significantly higher than in the benign group. This result indicates that serum OPN level may be a useful and cheap marker for the evaluation of thyroid nodules confirmed as Bethesda III. Further, it may reduce the need for unnecessary surgical procedures for these nodules.

This study has several limitations. Although, we intended to investigate serum OPN in patients with all types of thyroid cancers, due to the limited number of test kits and that the majority of patients were diagnosed with PTC, our results cannot be extrapolated to patients other than those with PTC. Nevertheless, considering the unavailability of molecular tests in many centers, serum OPN may be useful for PTC risk assessment of thyroid nodules confirmed as Bethesda III. Contrary to other studies, we did not find an association between serum OPN and aggressiveness, and prognosis (Bramwell 2006, Le *et al.* 2006, Conway *et al.* 2009, Likui *et al.* 2010, Liersch *et al.* 2012, Güttler *et al.* 2013, Wang *et al.* 2018, Zhang *et al.* 2020). Finally, there is a substantial overlap in the scattergram results between benign and malignant lesions.

In conclusion, high serum OPN levels may be helpful to guide the diagnosis of Bethesda III nodules as PTC. This way, serum OPN may help clinicians and surgeons to more reliably select patients with Bethesda III thyroid cytology for considering thyroidectomy. Therefore, the rate of unnecessary thyroidectomies in patients with thyroid nodules diagnosed cytologically as Bethesda III can be decreased. However, considering the limitations of our preliminary study, these data should be supported by prospective studies with larger study groups.

## Declaration of interest

The authors declare that there is no conflict of interest that could be perceived as prejudicing the impartiality of the research reported.

## Funding

Funding for this study was provided by a grant from the Scientific Research Project Coordination Unit of Necmettin Erbakan University (Project No. 161518021).

## Author contribution statement

T U K performed experiments, analyzed data, and wrote the paper. M K conceived the study and wrote the paper. İ K performed experiments.
